# Supplementary material for: Revealing the diversity of commensal corynebacteria from a single human skin site
Source: mSystems. 2025 Sep 25;10(10):e00459-25. doi: 10.1128/msystems.00459-25 (PMC12542688; doi:10.1128/msystems.00459-25)
Supplement: Supplemental figures and tables. — Fig. S1-S20; Tables S1-S7. [file msystems.00459-25-s0001.pdf]

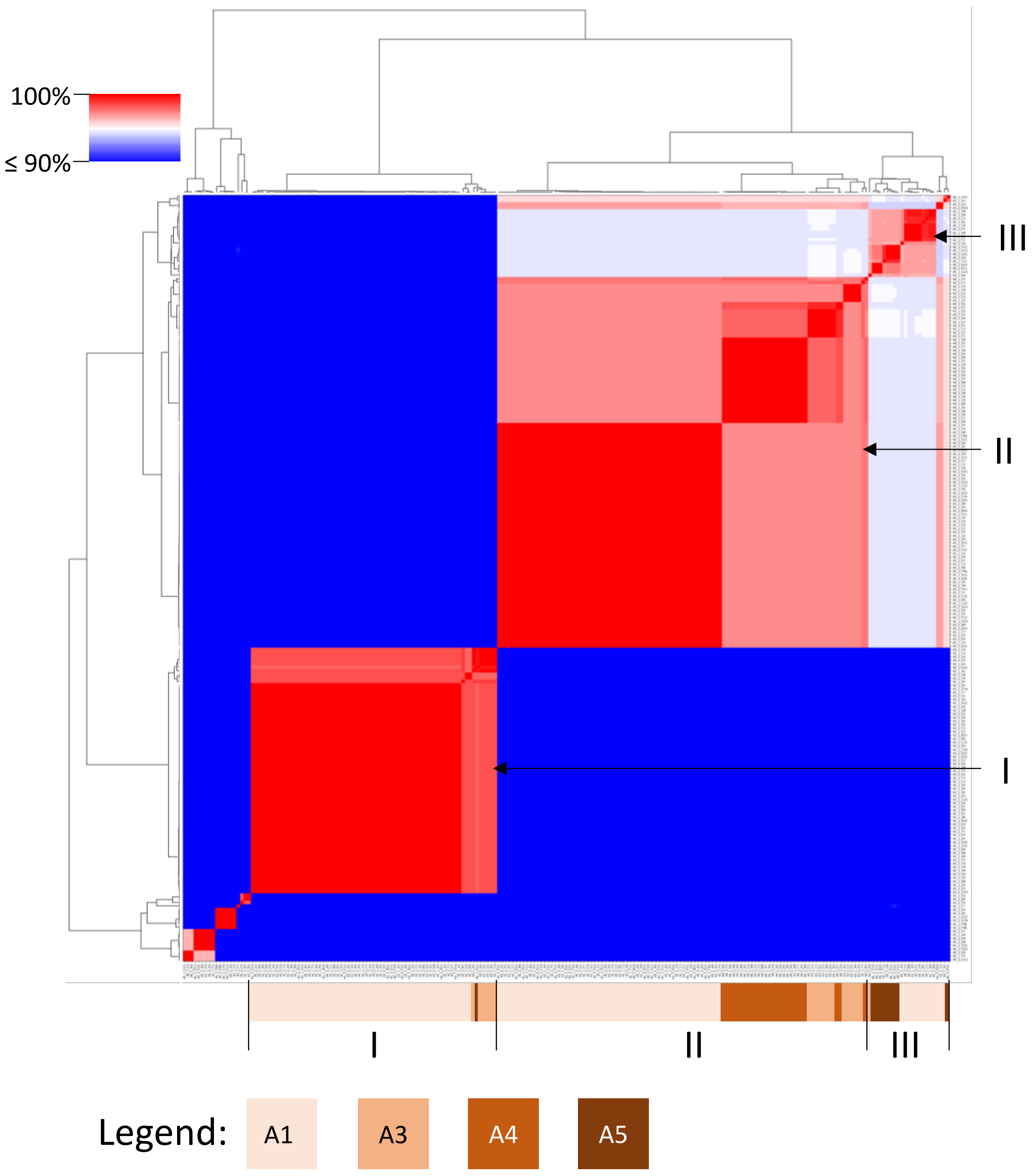

Supplementary Figure 1. Pairwise average nucleotide identities (ANI) of all isolates sequenced in this study. ANI values were placed onto a collective coloured pairwise matrix according to the colour scale depicted Three major groups of isolates were identified with I, II and III. Group I II and III isolates from each of the four individuals were denoted with the coloured bars as indicated in the legend.

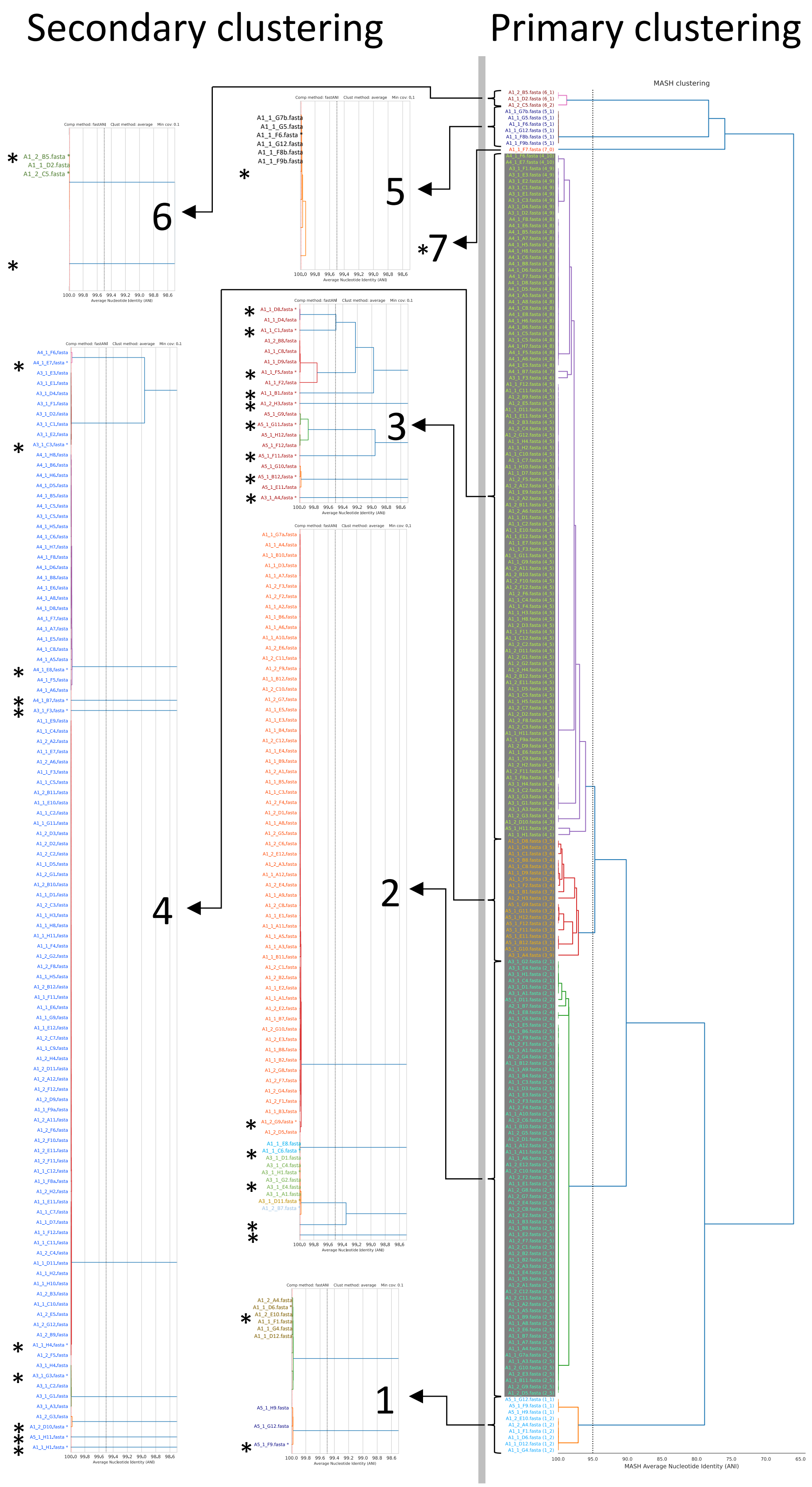

Supplementary Figure 2. Isolates were initially clustered by ANI to 7 primary clusters (species level) using MASH at a 95% threshold. A secondary clustering was performed on the isolates using fastANI at a 99.5% threshold to identify representatives from each cluster (labelled with *).

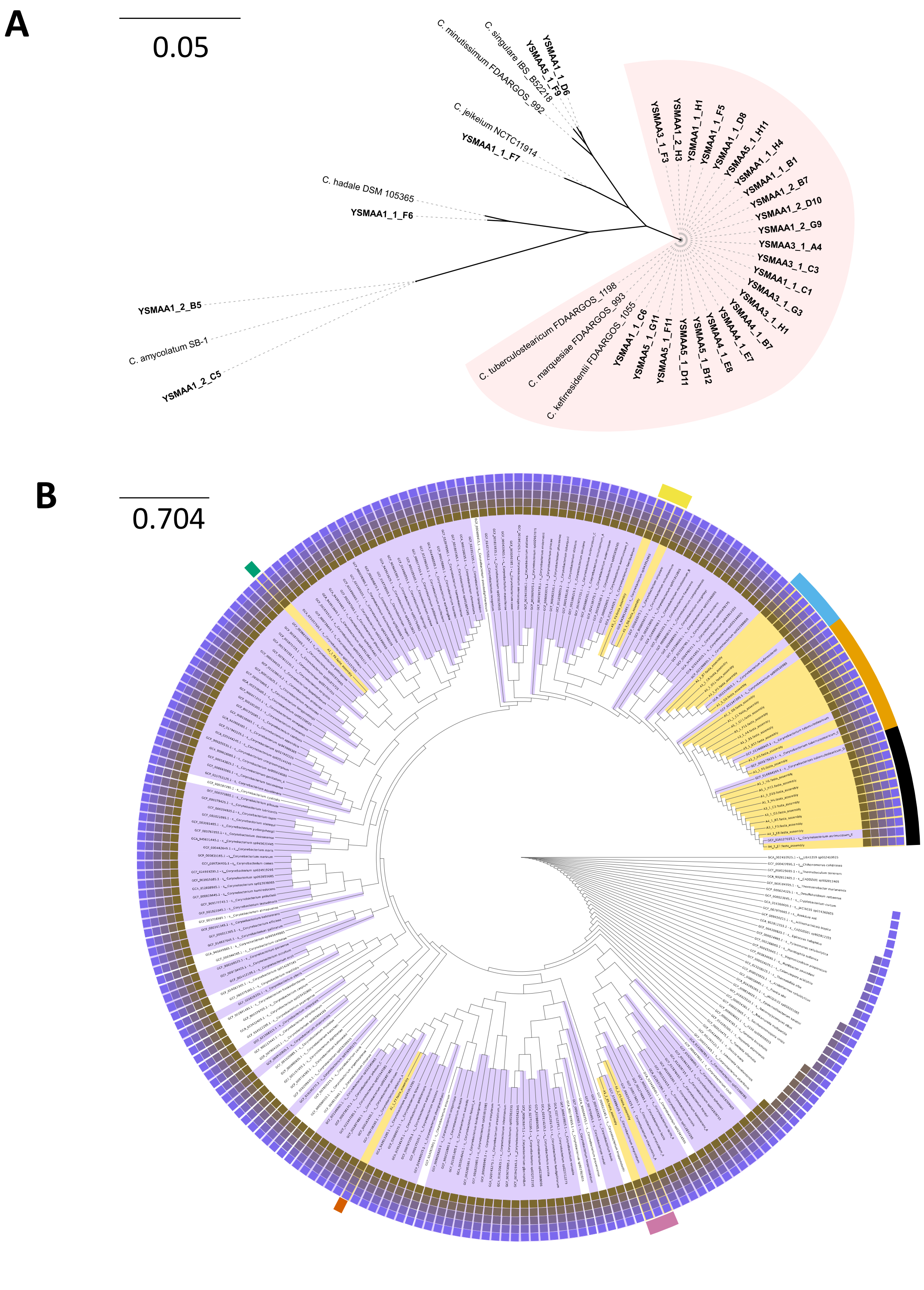

Supplementary Figure 3. Speciating the representative isolates. (A) The V1-V3 regions of the 16S rRNA sequences for each genome were aligned with those of the closest RefSeq genome BLASTn hits and plotted onto an unrooted tree. Branches within the red cloud share ≥ 99.8% identity. The tree was generated using PhyML and visualised on iTOL. (B) Genomes of the representative isolates (yellow) were compared to the genomes of the GTDB. 7 distinct groups of isolates were identified with coloured bars in the outermost ring (*C. kefirresidentii* – blue, *C. tuberculostearicum* – orange and *C. aurimucosum*_E/*C. marquesiae* – black, *C. axilliensis* – yellow, *C. gottingense* – green, *C. jamesii* – dark orange, *C. amycolatum* – magenta).

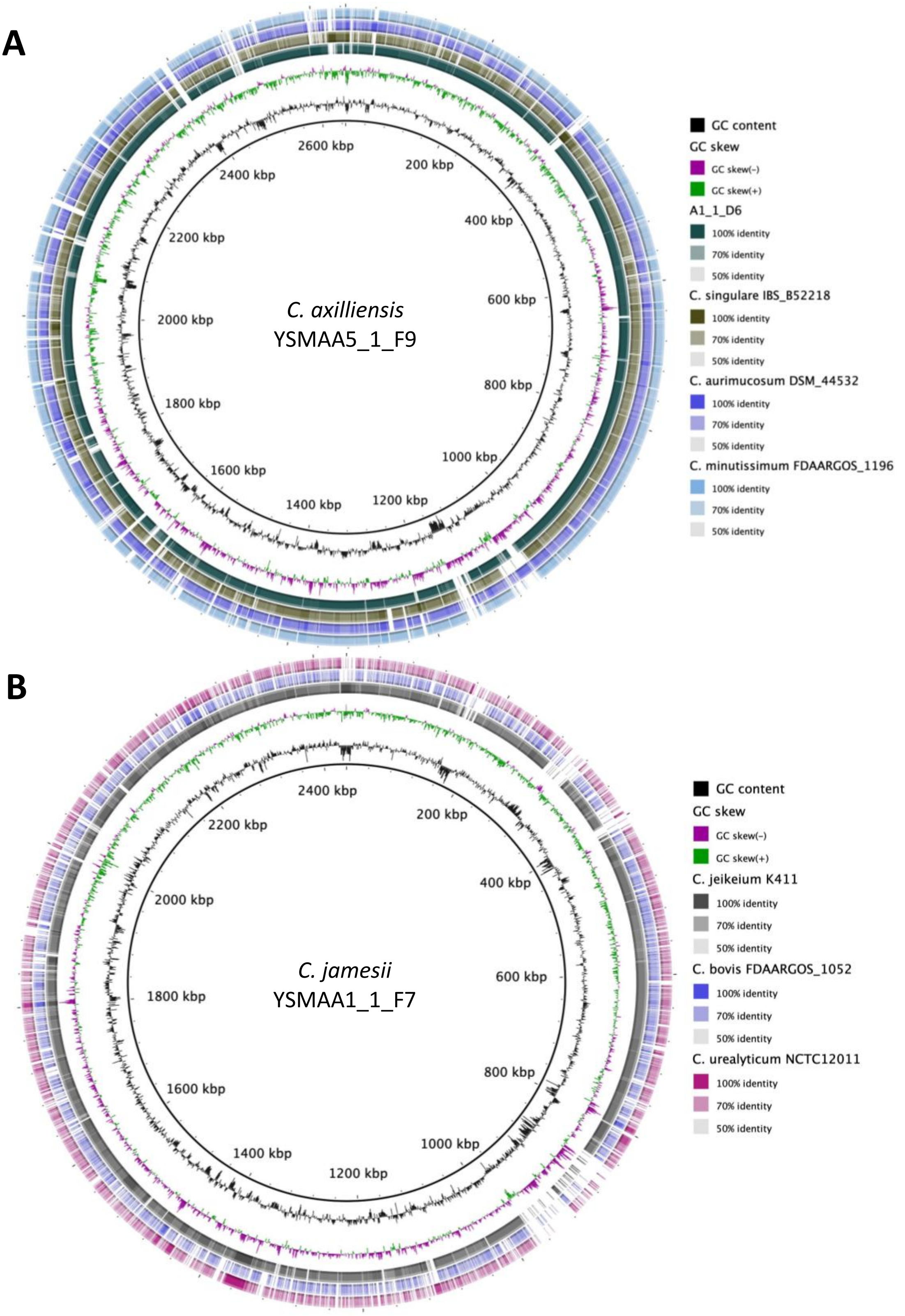

Supplementary Figure 4. Alignments of representatives from the two novel species *C. axilliensis* and *C. jamesii* against closest hits from GenBank. *C. axilliensis* YSMAA5_1_F9 and *C. jamesii* YSMAA1_1_F7 were used as the reference genomes on the plots. *C. axilliensis* YSMAA1_1_D6 was also included in (A) for comparison. Alignments and figures were generated using BRIG.

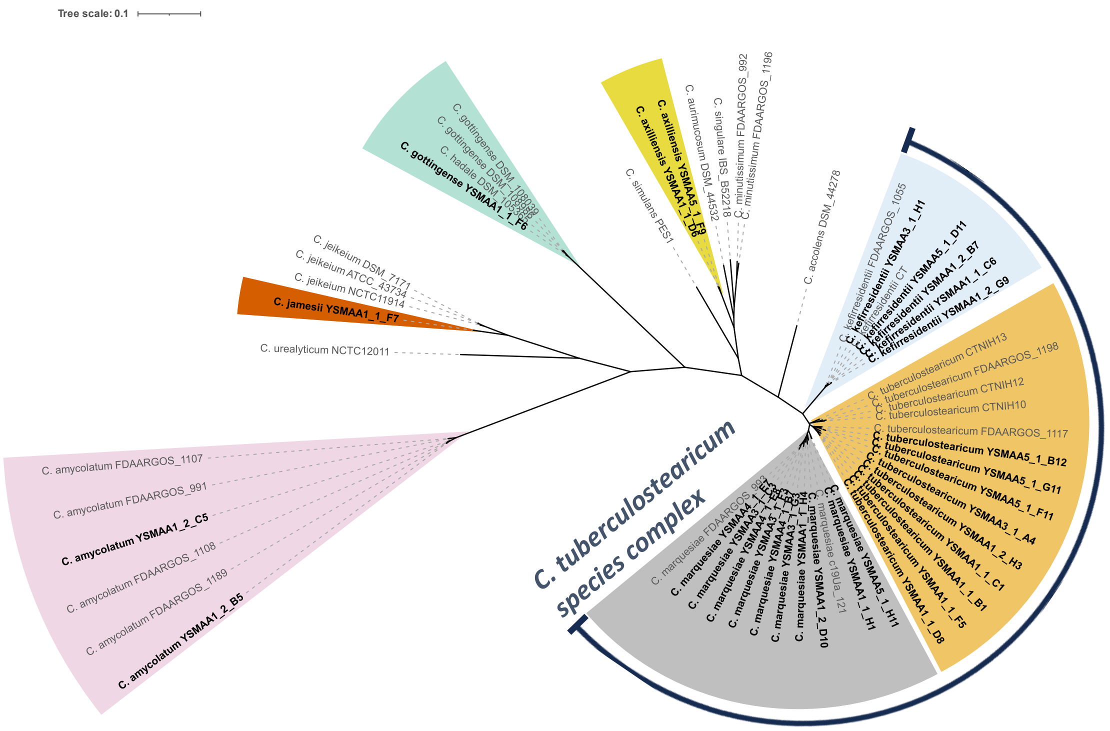

Supplementary Figure 5. A core genome alignment of the representative genomes (bold) and select RefSeq genomes was generated using Roary with a 80% BLASTp cutoff. The 7 species were identified using coloured wedges and the species within the *C. tuberculostearicum* species complex (grey, brown and blue wedges) were also distinguished. The tree was generated using PhyML and visualised on iTOL.

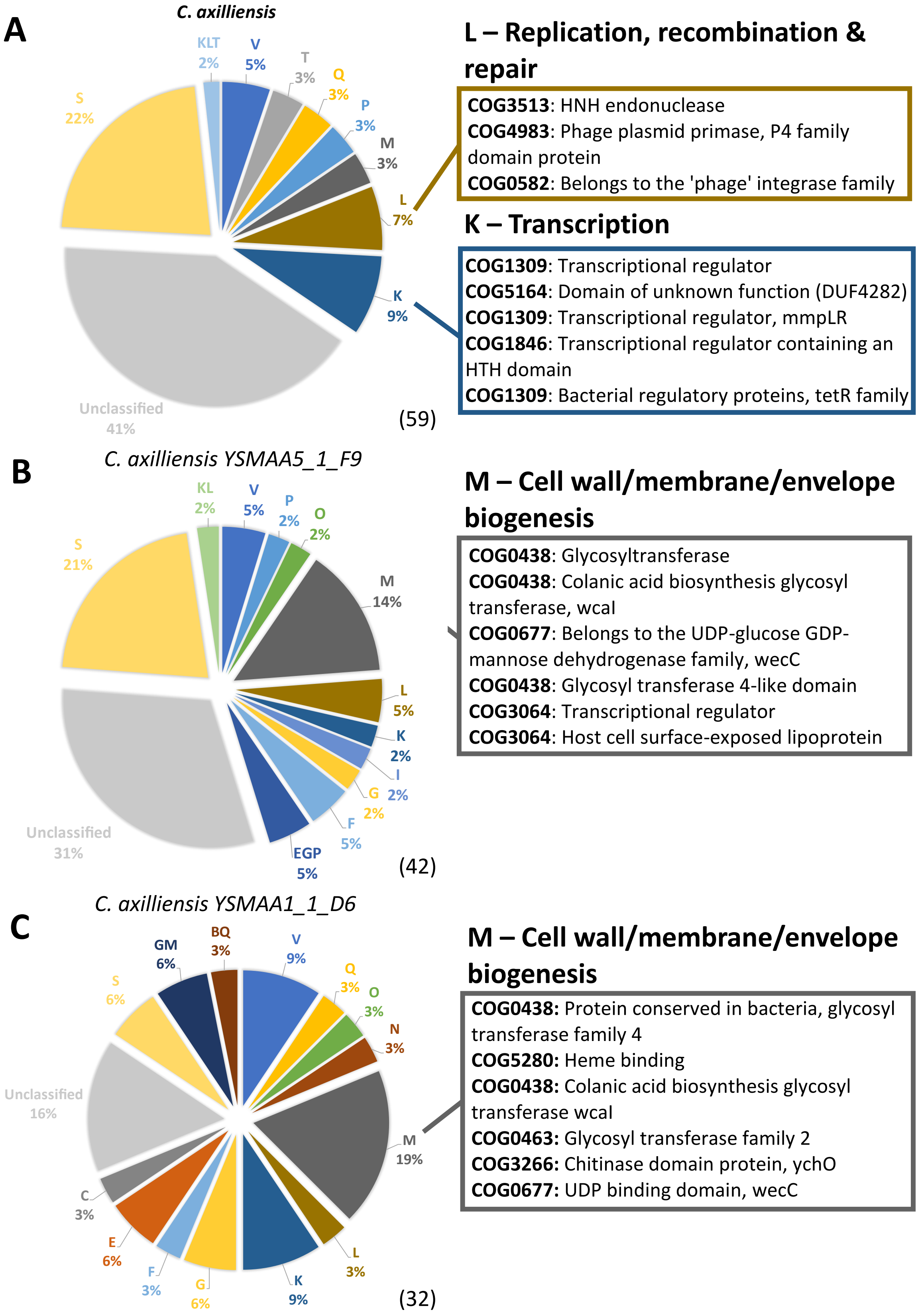

Supplementary Figure 6. COG classification of genes of the *C. axilliensis* (A) core genome and genes specific to (B) YSMAA5_1_F9 and (C) YSMAA1_1_D6. The percentage of genes within each COG classification is calculated against the total number of genes for each analysis. The genes of COG categories with the highest percentage and not unclassified or unknown (S) were shown.

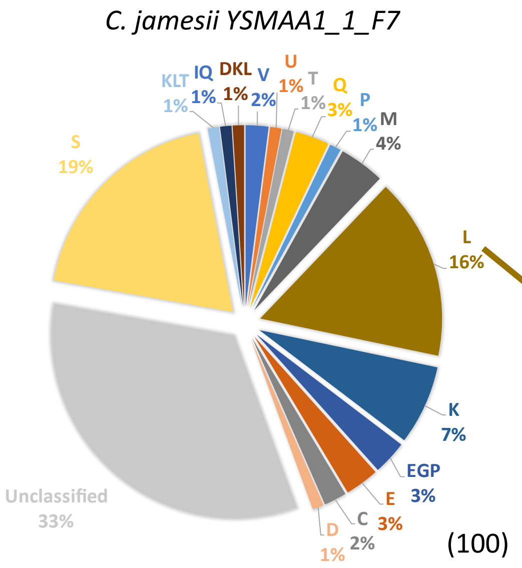

L – Replication, recombination and repair

COG0553: Helicase *X2*
COG5377: YqaJ-like viral recombinase domain
COG1961: Site-specific recombinases, DNA invertase Pin homologs
COG0507: Pfam PF08751 (AAA_30,TrwC,UvrD_C_2,Viral_helicase1) *X3*
COG3723: RecT family
COG0553: DEAD-like helicases superfamily
COG0305: Participates in initiation and elongation during chromosome replication, dnaB
COG0582: DNA integration
COG0847: EXOIII
COG1961: Site-specific recombinases, DNA invertase Pin homologs
COG0629: Single-strand binding protein family
COG2801: Integrase core domain
COG2801: InterPro IPR001584 (HTH_21,Terminase_5,rve)

Supplementary Figure 7. COG classification of genes of the *C. jamesii* YSMAA1_1_F7. The percentage of genes within each COG classification is calculated against the total number of genes for each analysis. The genes of COG categories with the highest percentage and not unclassified or unknown (S) were shown.

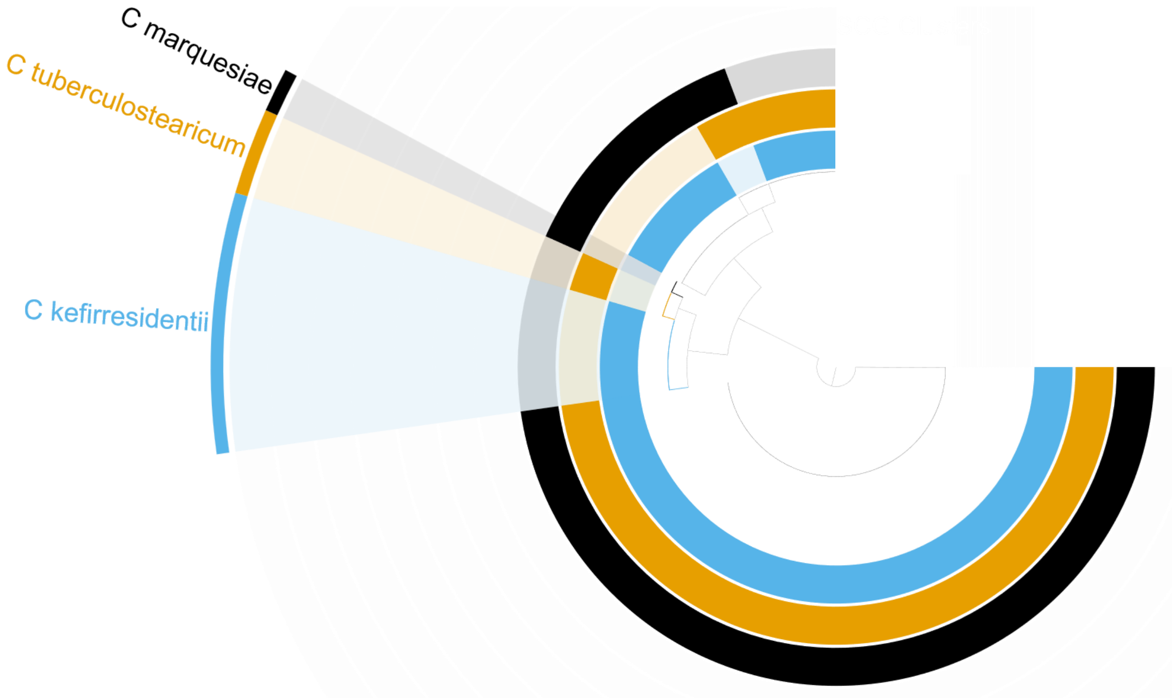

Supplementary Figure 8. Comparisons of the core genomes of species *C. marquesiae*, *C. tuberculostearicum* and *C. kefirresidentii* derived from the respective isolate genomes using anvi'o. Gene clusters specific to each species were highlighted using the coloured wedge.

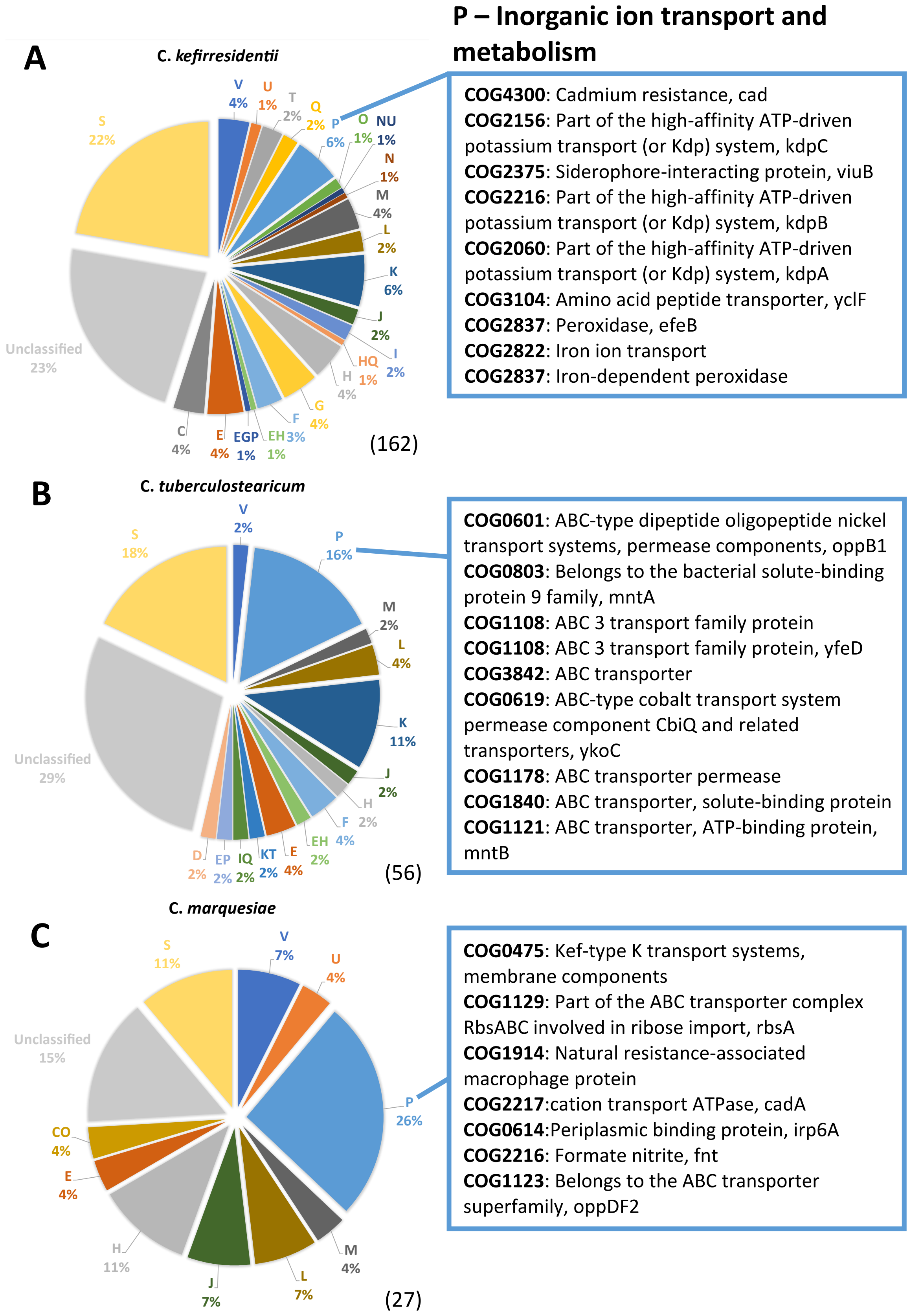

Supplementary Figure 9. COG classification of genes specific to (A) *C. kefirresidentii*, (B) *C. tuberculostearicum* and (C) *C. marquesiae*. The percentage of genes within each COG classification is calculated against the total number of genes for each analysis. The genes of COG categories with the highest percentage and not unclassified or unknown (S) were shown.

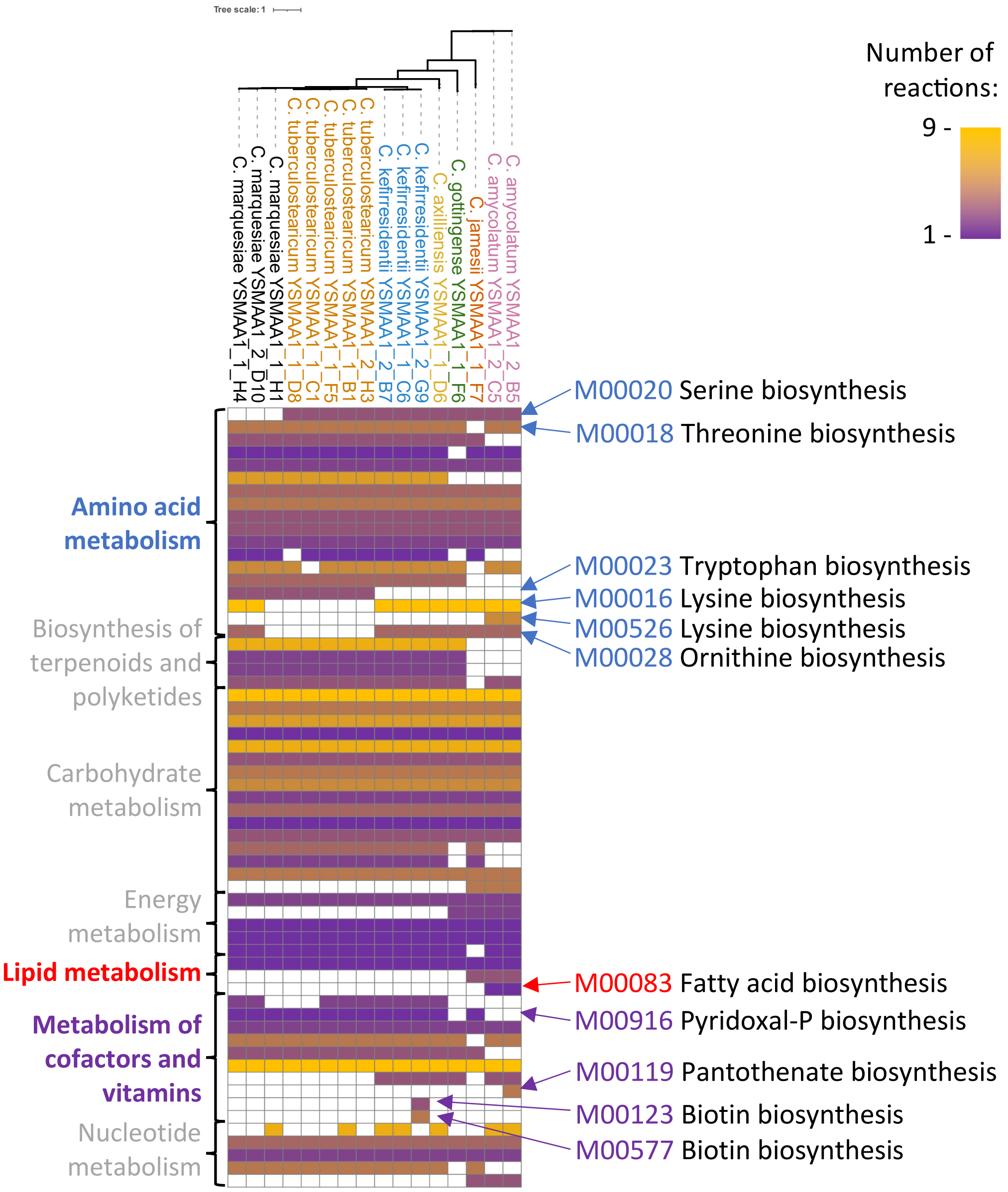

Supplementary Figure 10. Metabolic pathways in axillary corynebacteria of volunteer A1. Each KEGG module for each isolate were placed on a heatmap where the modules with the largest number of reactions are in yellow and lowest in purple with absent modules in white. Modules of interest were described on the right of the heatmap. KEGG modules were grouped according to the categories on the left of the heatmap. Isolates were grouped by species with a core genome tree depicting the variation.

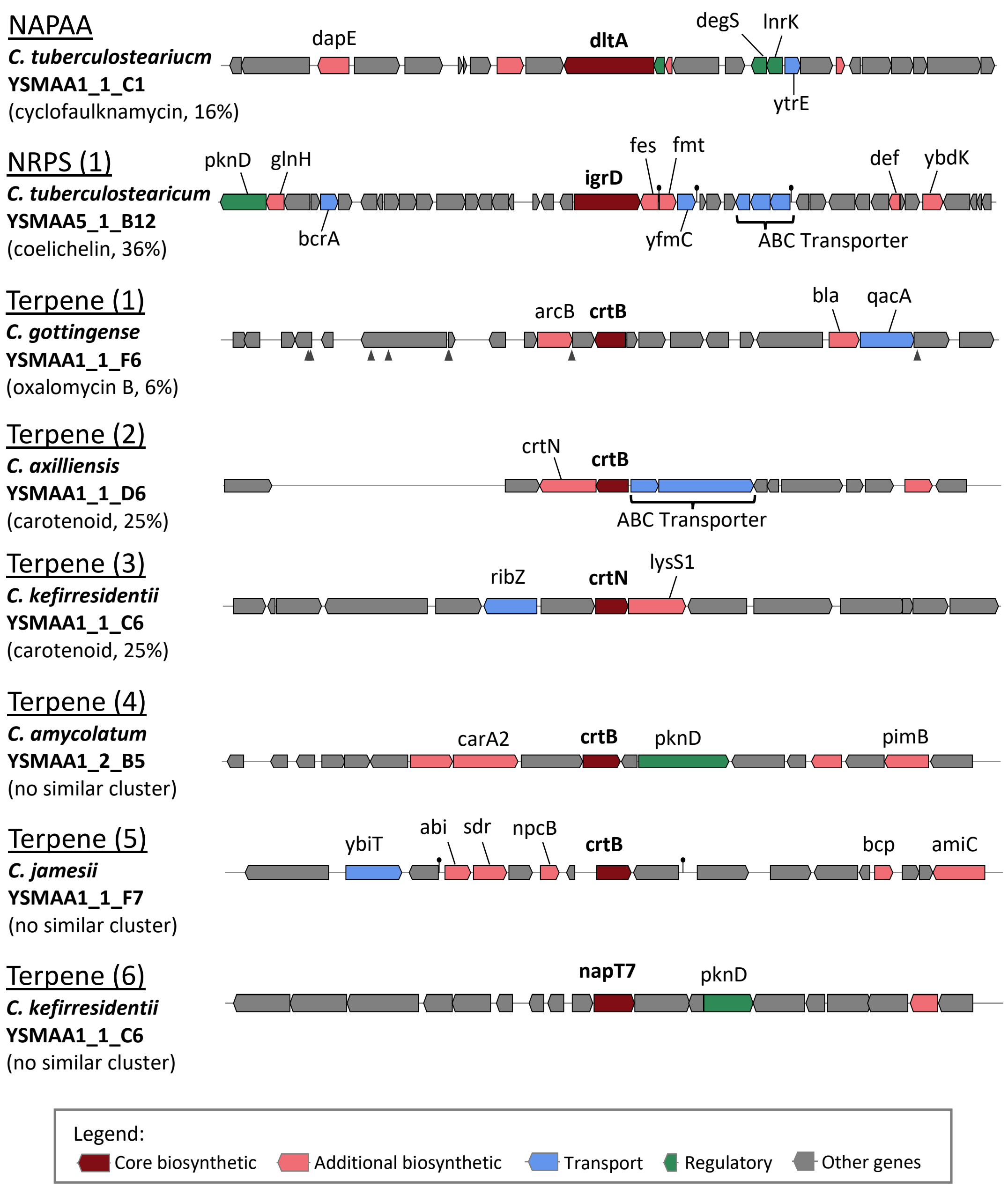

Supplementary Figure 11. Architectures of the 8 distinct putative biosynthetic gene clusters predicted using antiSMASH 7.

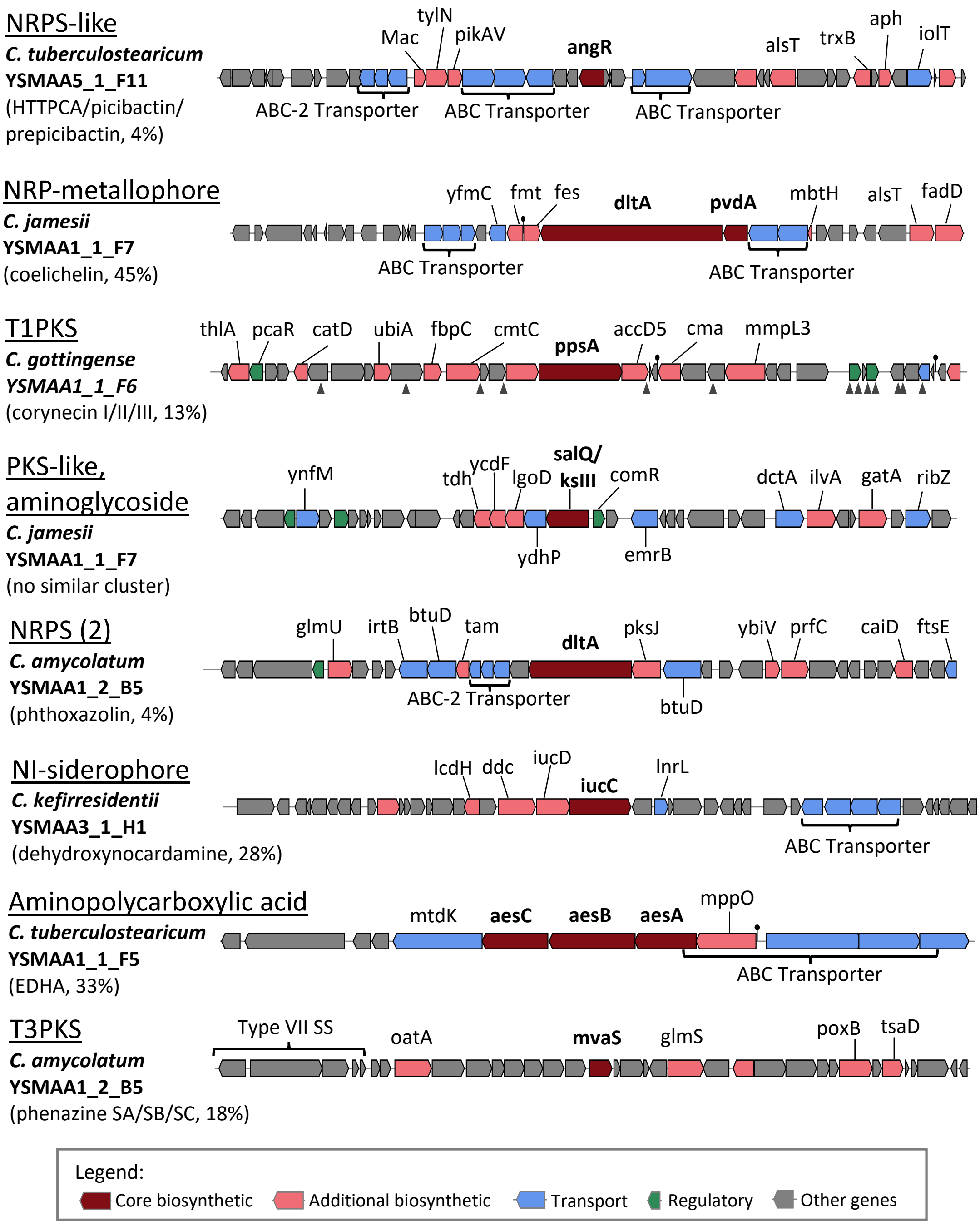

Supplementary Figure 12. Architectures of the 8 distinct putative biosynthetic gene clusters predicted using antiSMASH 7.

Region	Type	Most similar known cluster		Similarity
Region 1	terpene			
Region 2	NI-siderophore	dehydroxynocardamine	NRP	28%
Region 3	T1PKS			
Region 4	NAPAA	cyclofaulknamycin	Polyketide	16%
Region 5	terpene	carotenoid	Terpene	25%

C. kefirresidentii
A1_1_C6, A1_2_B7, A3_1_H1, A5_1_D11

Region	Type	Most similar known cluster		Similarity
Region 1	NRPS			
Region 2	T1PKS			
Region 3	NAPAA	ε-Poly-L-lysine	NRP	100%
Region 4	terpene	carotenoid	Terpene	25%

C. kefirresidentii
A1_2_G9

Region	Type	Most similar known cluster		Similarity
Region 1	terpene	carotenoid	Terpene	25%
Region 2	NAPAA	ε-Poly-L-lysine	NRP	100%
Region 3	T1PKS			

C. marquesiae A1_1_H4 also includes an aminopolycarboxylic acid cluster (most similar known cluster: EDHA, 33%)

C. marquesiae
A1_1_H1, A1_1_H4, A1_2_D10, A3_1_C3, A3_1_F3, A3_1_G3, A4_1_B7, A4_1_E7, A4_1_E8, A5_1_H11

Region	Type	Most similar known cluster		Similarity
Region 1	terpene	carotenoid	Terpene	25%
Region 2	NAPAA	cyclofaulknamycin	Polyketide	12%

C. tuberculostearicum
A3_1_A4

Region	Type	Most similar known cluster		Similarity
Region 1	T1PKS			
Region 2	NRPS	coelichelin	NRP	36%
Region 3	NAPAA	ε-Poly-L-lysine	NRP	100%
Region 4	terpene	carotenoid	Terpene	25%
Region 5	NRPS-like	HTTPCA/prepiscibactin/piscibactin	NRP+Polyketide	4%
Region 6	NRPS	glycopeptidolipid	NRP	12%

C. tuberculostearicum
A5_1_B12, A5_1_F11

Region	Type	Most similar known cluster		Similarity
Region 1	T1PKS			
Region 2	NRPS	coelichelin	NRP	36%
Region 3	NAPAA	ε-Poly-L-lysine	NRP	100%
Region 4	terpene	carotenoid	Terpene	25%
Region 5	NRPS	glycopeptidolipid	NRP	20%

C. tuberculostearicum
A1_2_H3, A5_1_G11

Region	Type	Most similar known cluster		Similarity
Region 1	NRPS	glycopeptidolipid	NRP	20%
Region 2	T1PKS			
Region 3	NAPAA	cyclofaulknamycin	Polyketide	12%
Region 4	terpene	carotenoid	Terpene	25%

C. tuberculostearicum
A1_1_B1, A1_1_C1, A1_1_D8, A1_1_F5

C. tuberculostearicum A1_1_F5 also includes an aminopolycarboxylic acid cluster (most similar known cluster: EDHA, 33%)

Supplementary Figure 13. The distribution of putative biosynthetic gene clusters in the *C. tuberculostearicum* species complex predicted using antiSMASH 7.

Region	Type	Most similar known cluster		Similarity
Region 1	NRPS	phthoxazolin	NRP+Polyketide	4%
Region 2	terpene			
Region 3	T3PKS	phenazine SA/phenazine SB/phenazine SC	Alkaloid	18%
Region 4	NAPAA	ε-Poly-L-lysine	NRP	100%

C. amycolatum
A1_2_B5,
A1_2_C5

Region	Type	Most similar known cluster		Similarity
Region 1	T1PKS	corynecin III/corynecin I/corynecin II	Other	13%
Region 2	terpene	oxalomycin B	NRP+Polyketide	6%
Region 3	NRPS	coelibactin	NRP	18%

C. gottingense
A1_1_F6

Region	Type	Most similar known cluster		Similarity
Region 1	T1PKS			
Region 2	NAPAA	ε-Poly-L-lysine	NRP	100%
Region 3	terpene	carotenoid	Terpene	25%

C. axilliensis
A1_1_D6, A5_1_F9

Region	Type	Most similar known cluster		Similarity
Region 1	PKS-like, amglyccycl			
Region 2	terpene			
Region 3	NRP-metallophore, NRPS	coelichelin	NRP	45%
Region 4	NAPAA	ε-Poly-L-lysine	NRP	100%
Region 5	T1PKS			

C. jamesii
A1_1_F7

Supplementary Figure 14. The distribution of putative biosynthetic gene clusters in the *C. amycolatum, C. gottingense, C. axilliensis and C jamesii* isolates predicted using antiSMASH 7.

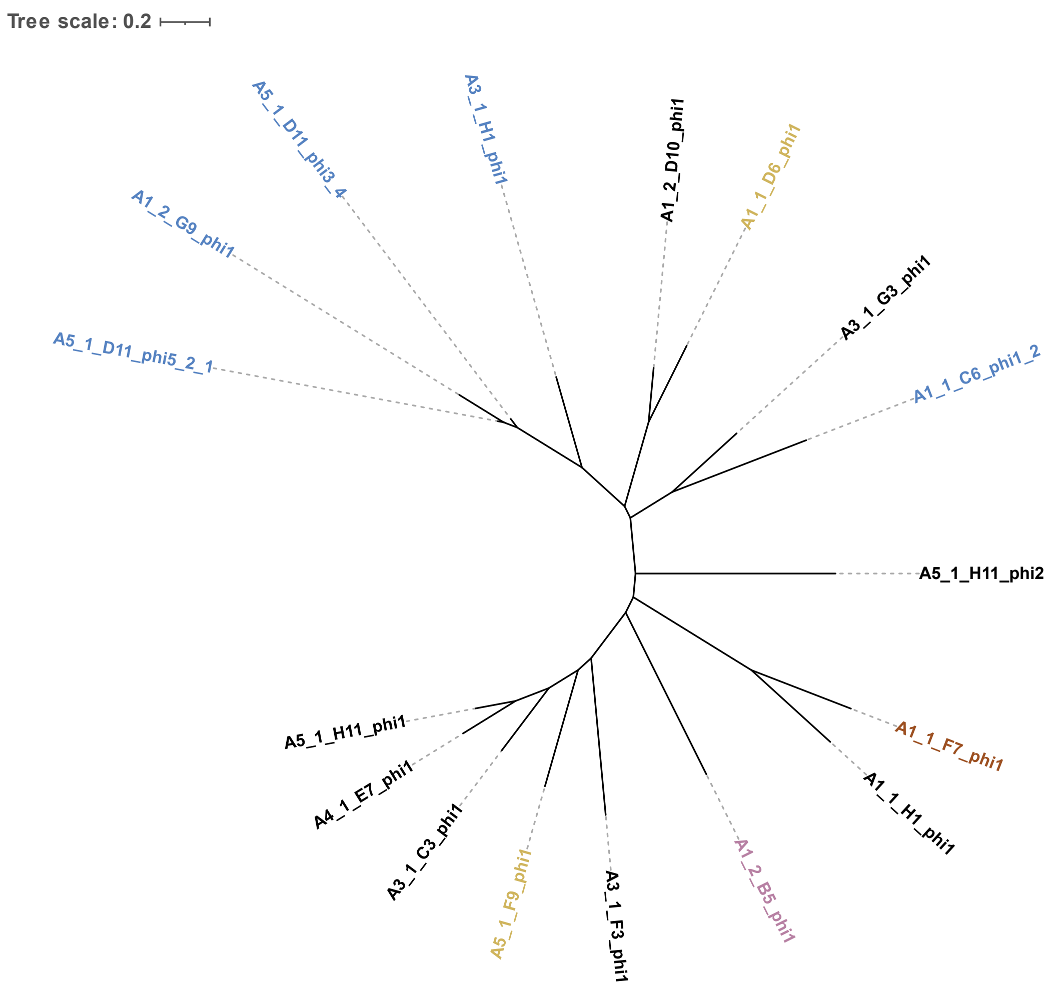

Supplementary Figure 15. None of the putative prophages were completely identical to each other. Putative prophages were aligned and compared using an unrooted tree generated with PhyML and visualised on iTOL. Putative prophages were coloured by the species of the source isolate (Blue: *C. kefirresidentii*, Black: *C. marquesiae*, Gold: *C. axilliensis*, Orange: *C. jamesii* and Pink: *C. amycolatum*).

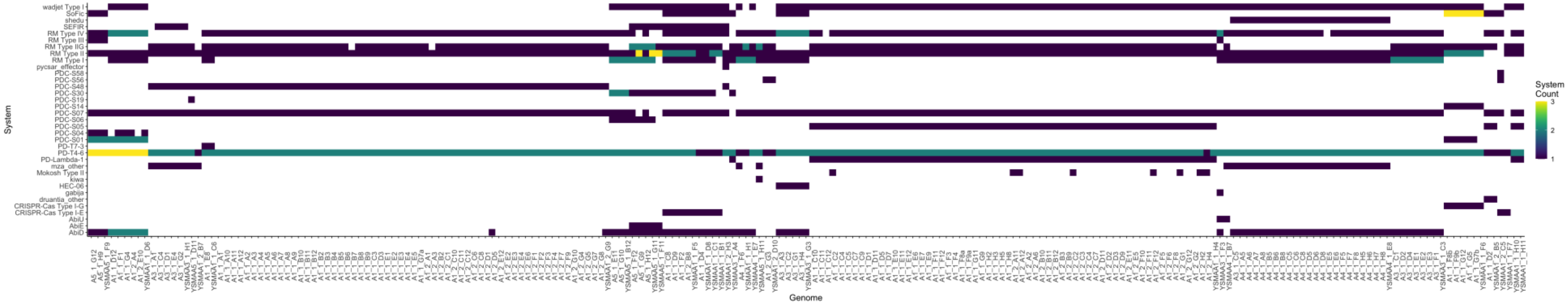

Supplementary Figure 16. Global PADLOC analysis reveals profiles of phage defence systems are generally conserved within drep secondary clusters.

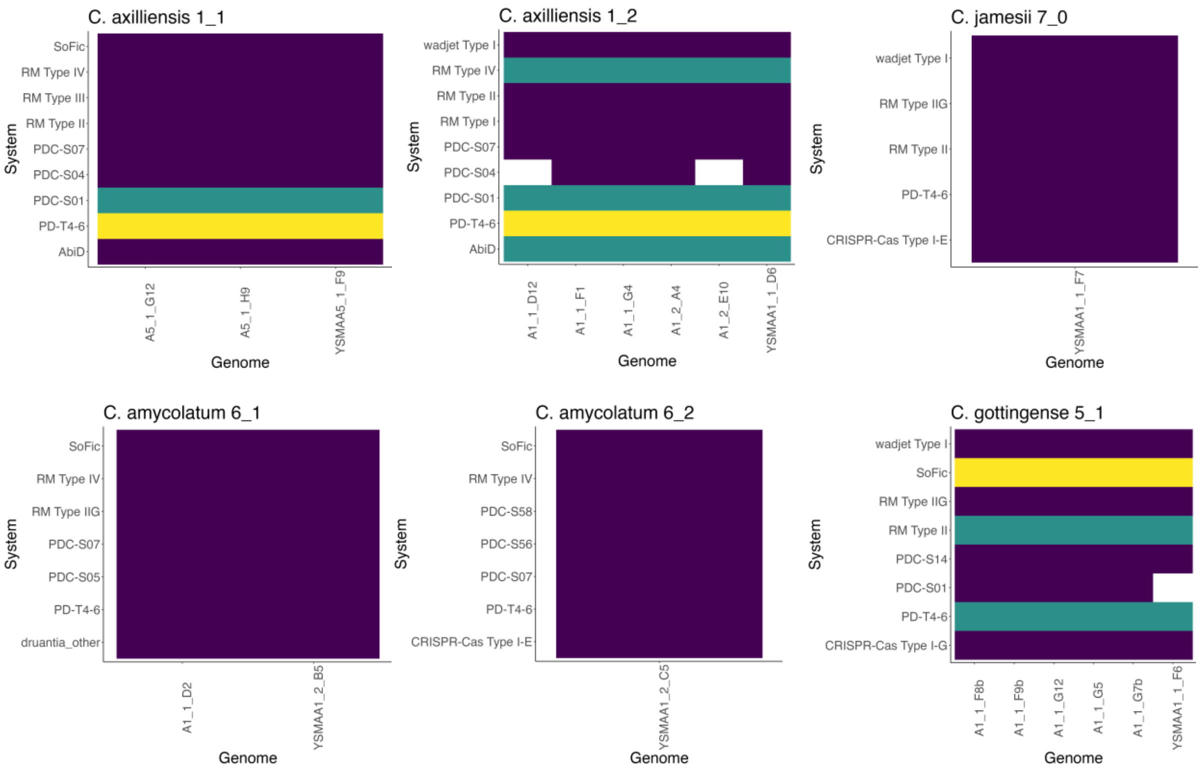

Supplementary Figure 17. Phage defence systems found in drep secondary clusters of (A-B) *C. axilliensis*, (C) *C. jamesii*, (D-E) *C. amycolatum* and (F) *C. gottingense*. The heatmap describes the number of each identified system with (Yellow: 3, Green: 2, Purple: 1 and White: 0).

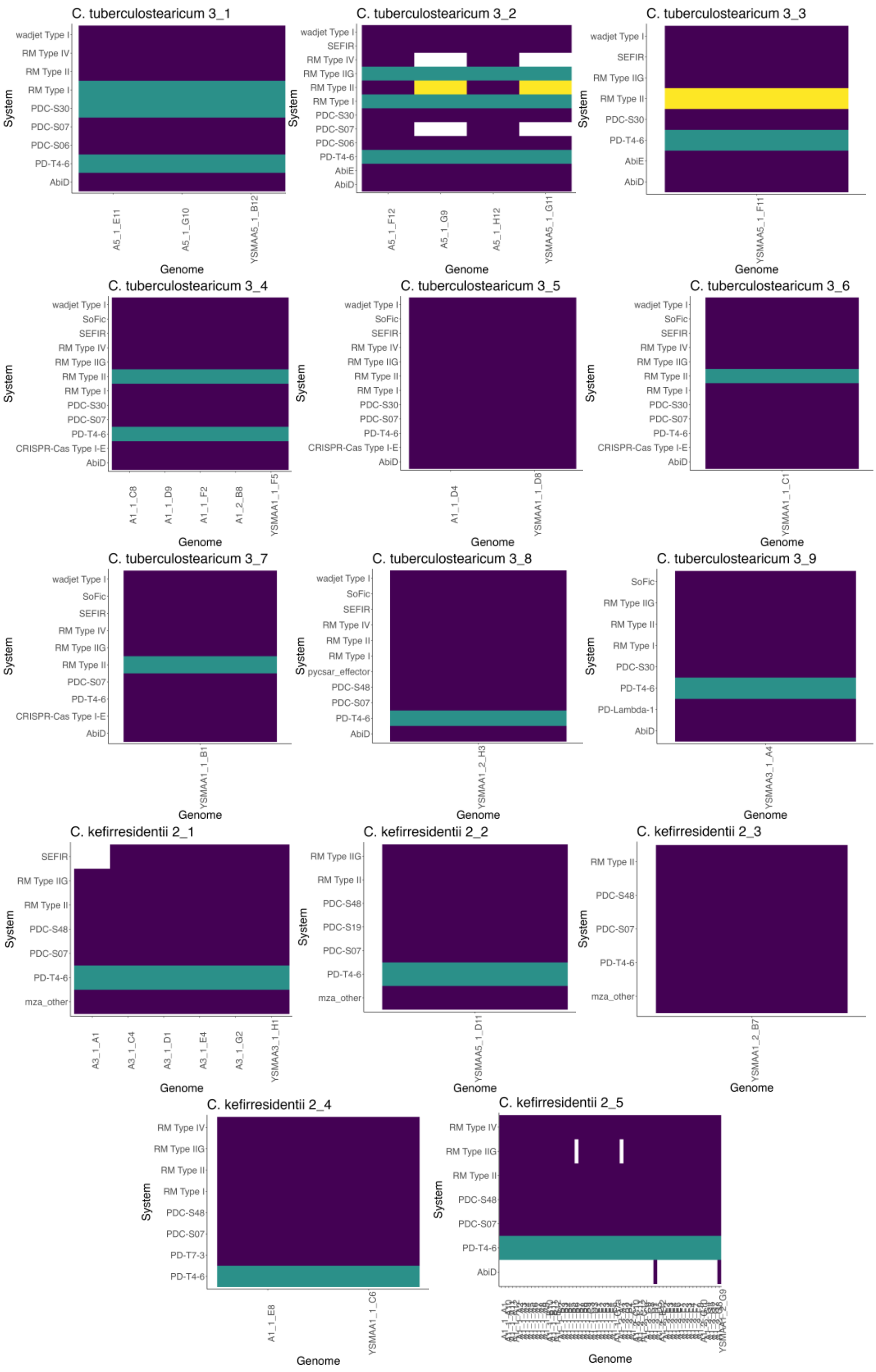

Supplementary Figure 18. Phage defence systems found in drep secondary clusters of (A-I) *C. tuberculostearicum* and (J-N) *C. kefirresidentii*. The heatmap describes the number of each identified system with (Yellow: 3, Green: 2, Purple: 1 and White: 0).

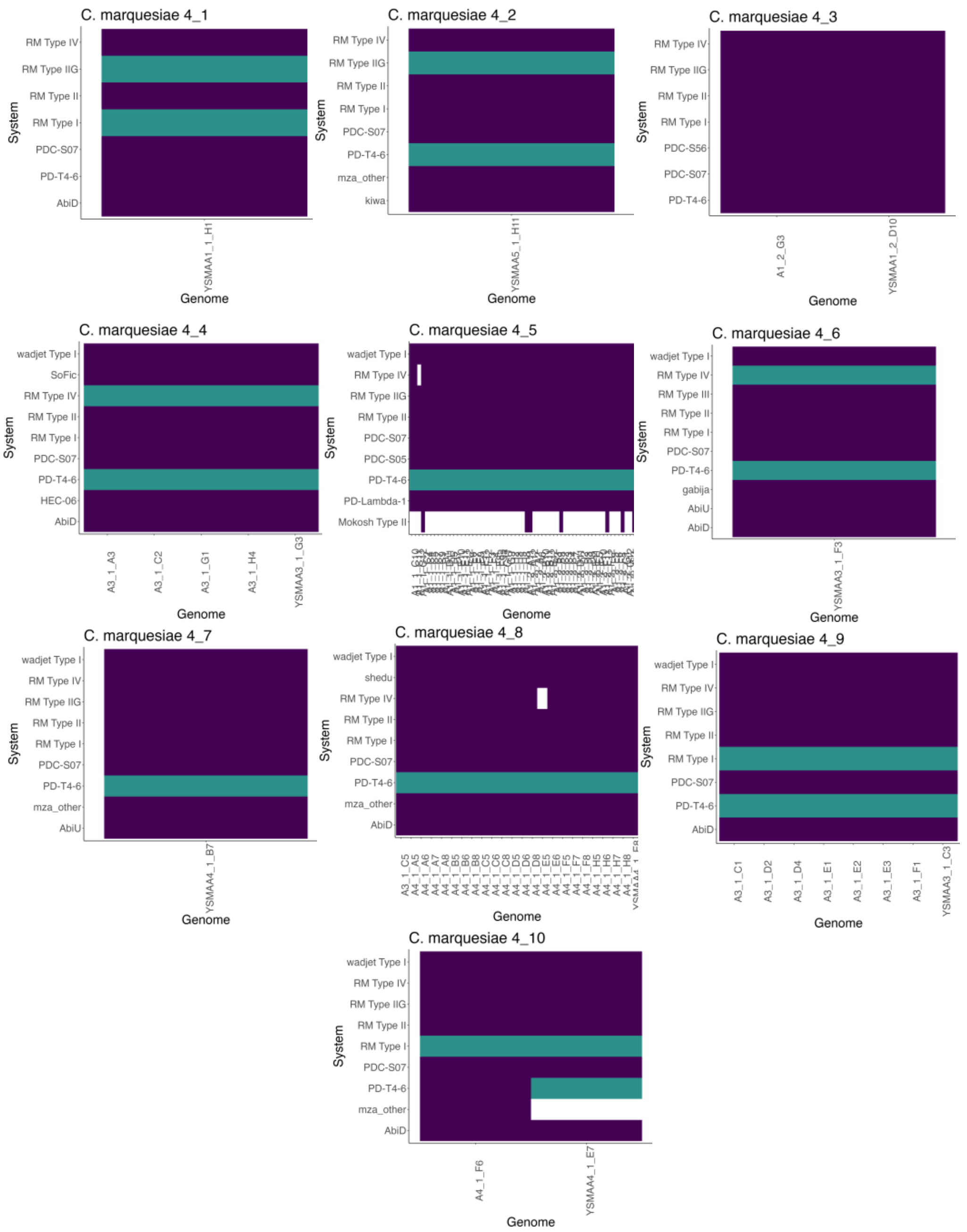

Supplementary Figure 19. Phage defence systems found in drep secondary clusters of (A-J) *C. marquesiae*. The heatmap describes the number of each identified system with (Yellow: 3, Green: 2, Purple: 1 and White: 0).

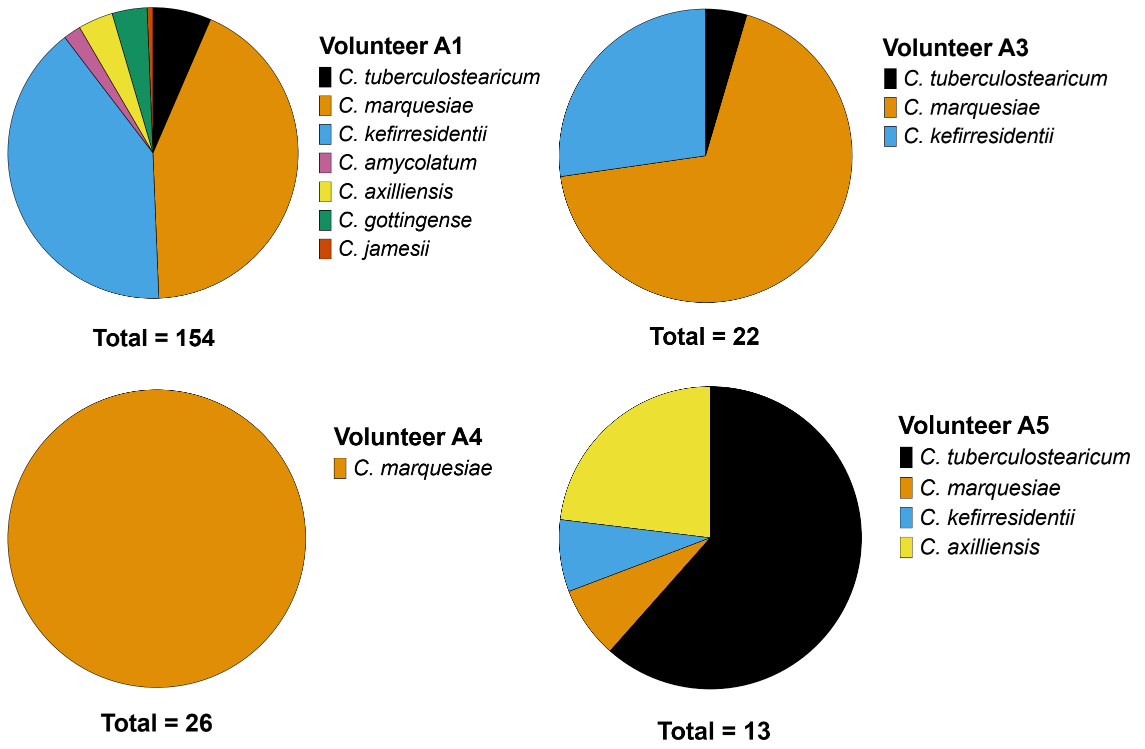

Supplementary Figure 20. The proportion of the total number of isolates derived from each of the 4 volunteers which bin to the 7 identified species (*C. tuberculostearicum*, *C. marquesiae*, *C. kefirresidentii*, *C. amycolatum*, *C. axilliensis*, *C. gottingense* and *C. jamesii*)

Isolate	Classification	FastANI Reference	FastANI Reference Radius	FastANI ANI	FastANI Alignment Fraction	Other Related References	MSA AA Percent	RED Value
YSMAA1_2_B5	g__Corynebacterium;s__Corynebacterium amycolatum	GCF_016889425.1	95	99.49	0.978	GCF_000173655.1	96.62	-
YSMAA1_2_C5	g__Corynebacterium;s__Corynebacterium amycolatum	GCF_016889425.1	95	98.55	0.959	GCF_000173655.1	96.6	-
YSMAA1_1_F6	g__Corynebacterium;s__Corynebacterium gottingense	GCF_003693265.1	95	97.62	0.927	GCF_001831515.1	95.65	-
YSMAA1_1_D6^	g__Corynebacterium;s__Corynebacterium sp943912665	GCA_943912665.1	95	96.42	0.928	GCF_000833575.1	96.17	-
YSMAA5_1_F9^	g__Corynebacterium;s__Corynebacterium sp943912665	GCA_943912665.1	95	97.51	0.952	GCF_000833575.1	95.73	-
YSMAA1_1_F7^	g__Corynebacterium;s__Corynebacterium sp943913395	GCA_943913395.1	95	96.82	0.953	GCF_000738175.1	95.39	-
YSMAA1_1_C6	g__Corynebacterium;s__Corynebacterium kefirresidentii	GCA_002154655.1	95	96.37	0.901	GCF_022288805.1	95.89	-
YSMAA1_2_B7	g__Corynebacterium;s__Corynebacterium kefirresidentii	GCA_002154655.1	95	96.43	0.9	GCF_022288805.1	95.85	-
YSMAA1_2_G9	g__Corynebacterium;s__Corynebacterium kefirresidentii	GCA_002154655.1	95	96.42	0.899	GCF_022288805.1	95.15	-
YSMAA3_1_H1	g__Corynebacterium;s__Corynebacterium kefirresidentii	GCA_002154655.1	95	96.5	0.908	GCF_022288805.1	95.65	-
YSMAA5_1_D11	g__Corynebacterium;s__Corynebacterium kefirresidentii	GCA_002154655.1	95	96.31	0.903	GCF_022288805.1	96.17	-
YSMAA1_1_C1	g__Corynebacterium;s__Corynebacterium tuberculostearicu	GCF_013408445.1	96.3332	96.4	0.92	GCF_013408445.1	96.21	-
YSMAA1_1_D8	g__Corynebacterium;s__Corynebacterium tuberculostearicu	GCF_013408445.1	96.3332	96.39	0.928	GCF_000175635.1	96.23	-
YSMAA1_1_F5	g__Corynebacterium;s__Corynebacterium tuberculostearicu	GCF_013408445.1	96.3332	96.42	0.923	GCF_000175635.1	96.21	-
YSMAA1_2_H3	g__Corynebacterium;s__Corynebacterium tuberculostearicu	GCF_013408445.1	96.3332	96.49	0.938	GCF_016894265.1	96.15	-
YSMAA3_1_A4	g__Corynebacterium;s__Corynebacterium tuberculostearicum	GCF_013408445.1	96.3332	96.33	0.924	GCF_013408445.1	92.63	-
YSMAA5_1_B12	g__Corynebacterium;s__Corynebacterium tuberculostearicu	GCF_013408445.1	96.3332	96.61	0.903	GCF_000175635.1	95.83	-
YSMAA5_1_G11	g__Corynebacterium;s__Corynebacterium tuberculostearicum_	GCF_000175635.1	96.3332	96.47	0.934	GCF_013408445.1	94.2	-
YSMAA1_1_B1*	g__Corynebacterium;s__	-	-	-	-	GCF_013408445.1	96.21	0.99902
YSMAA5_1_F11*	g__Corynebacterium;s__	-	-	-	-	GCF_000175635.1	96.01	0.999
YSMAA1_1_H1	g__Corynebacterium;s__Corynebacterium aurimucosum_E	GCF_016127015.1	95	95.58	0.911	GCF_013408445.1	95.37	-
YSMAA1_1_H4	g__Corynebacterium;s__Corynebacterium aurimucosum_E	GCF_016127015.1	95	97.24	0.94	GCF_022347205.1	94.9	-
YSMAA1_2_D10	g__Corynebacterium;s__Corynebacterium aurimucosum_E	GCF_016127015.1	95	96.54	0.91	GCF_022347205.1	95.75	-
YSMAA3_1_C3	g__Corynebacterium;s__Corynebacterium aurimucosum_E	GCF_016127015.1	95	99.33	0.97	GCF_000175635.1	95.47	-
YSMAA3_1_F3	g__Corynebacterium;s__Corynebacterium aurimucosum_E	GCF_016127015.1	95	98.05	0.935	GCF_000175635.1	95.73	-
YSMAA3_1_G3	g__Corynebacterium;s__Corynebacterium aurimucosum_E	GCF_016127015.1	95	97.35	0.92	GCF_000175635.1	95.45	-
YSMAA4_1_B7	g__Corynebacterium;s__Corynebacterium aurimucosum_E	GCF_016127015.1	95	97.52	0.941	GCF_000175635.1	95.41	-
YSMAA4_1_E7	g__Corynebacterium;s__Corynebacterium aurimucosum_E	GCF_016127015.1	95	99.62	0.98	GCF_000175635.1	96.15	-
YSMAA4_1_E8	g__Corynebacterium;s__Corynebacterium aurimucosum_E	GCF_016127015.1	95	98.31	0.949	GCF_022347205.1	95.83	-
YSMAA5_1_H11	g__Corynebacterium;s__Corynebacterium aurimucosum_E	GCF_016127015.1	95	95.6	0.915	GCF_013408445.1	95.77	-

Supplementary Table 1. Representative genomes were compared on GTDB-tk to identify the closest species on the Genome Taxonomy Database (Classification) using FastANI with the threshold for each classification defined on the "FastANI Reference Radius" tab. Isolates are coloured by classification groups.

Species	Strain (ANI ≥ 99.5%)	Clones	Representative isolate
C. axilliensis	1_2	6	YSMAA1_1_D6
C. kefirresidentii	2_3	1	YSMAA1_2_B7
C. kefirresidentii	2_4	2	YSMAA1_1_C6
C. kefirresidentii	2_5	59	YSMAA1_2_G9
C. tuberculostearicum	3_4	5	YSMAA1_1_F5
C. tuberculostearicum	3_5	2	YSMAA1_1_D8
C. tuberculostearicum	3_6	1	YSMAA1_1_C1
C. tuberculostearicum	3_7	1	YSMAA1_1_B1
C. tuberculostearicum	3_8	1	YSMAA1_2_H3
C. marquesiae	4_1	1	YSMAA1_1_H1
C. marquesiae	4_3	2	YSMAA1_2_D10
C. marquesiae	4_5	63	YSMAA1_1_H4
C. gottingense	5_1	6	YSMAA1_1_F6
C. amycolatum	6_1	2	YSMAA1_2_B5
C. amycolatum	6_2	1	YSMAA1_2_C5
C. jamesii	7_0	1	YSMAA1_1_F7

Supplementary Table 2. Summary of *Corynebacterium* isolates from volunteer A1. The initial pool of 154 isolates were sorted into specific species (ANI ≥ 95%) and strains (ANI ≥ 99.5%) using drep. Species were identified using GTDB-tk. Number of clones and the representative isolates for each strain were included.

Species	Strain (ANI ≥ 99.5%)	Clones	Representative isolate
C. kefirresidentii	2_1	6	YSMAA3_1_H1
C. tuberculostearicum	3_9	1	YSMAA3_1_A4
C. marquesiae	4_4	5	YSMAA3_1_G3
C. marquesiae	4_6	1	YSMAA3_1_F3
C. marquesiae	4_8	1	YSMAA4_1_E8
C. marquesiae	4_9	8	YSMAA3_1_C3

Supplementary Table 3. Summary of *Corynebacterium* isolates from volunteer A3. The initial pool of 22 isolates were sorted into specific species (ANI ≥ 95%) and strains (ANI ≥ 99.5%) using drep. Species were identified using GTDB-tk. Number of clones and the representative isolates for each strain were included.

Species	Strain (ANI ≥ 99.5%)	Clones	Representative isolate
C. marquesiae	4_7	1	YSMAA4_1_B7
C. marquesiae	4_8	23	YSMAA4_1_E8
C. marquesiae	4_10	2	YSMAA4_1_E7

Supplementary Table 4. Summary of *Corynebacterium* isolates from volunteer A4. The initial pool of 26 isolates were sorted into specific species (ANI ≥ 95%) and strains (ANI ≥ 99.5%) using drep. Species were identified using GTDB-tk. Number of clones and the representative isolates for each strain were included.

Species	Strain (ANI ≥ 99.5%)	Clones	Representative isolate
C. axilliensis	1_1	3	YSMAA5_1_F9
C. kefirresidentii	2_2	1	YSMAA5_1_D11
C. tuberculostearicum	3_1	3	YSMAA5_1_B12
C. tuberculostearicum	3_2	4	YSMAA5_1_G11
C. tuberculostearicum	3_3	1	YSMAA5_1_F11
C. marquesiae	4_2	1	YSMAA5_1_H11

Supplementary Table 5. Summary of *Corynebacterium* isolates from volunteer A5. The initial pool of 13 isolates were sorted into specific species (ANI ≥ 95%) and strains (ANI ≥ 99.5%) using drep. Species were identified using GTDB-tk. Number of clones and the representative isolates for each strain were included.

Isolate	1	2	3	4	5	6
YSMAA1_1_C1	**erm(X)**, U21300, [lincomycin, clindamycin, erythromycin, quinupristin , pristinamycin ia, virginiamycin s]					
YSMAA1_1_D6	**erm(X)**, X51472, [lincomycin, clindamycin, erythromycin, quinupristin , pristinamycin ia, virginiamycin s]					
YSMAA1_1_F6	**erm(X)**, X51472, [lincomycin, clindamycin, erythromycin, quinupristin , pristinamycin ia, virginiamycin s]					
YSMAA1_1_H4	**aac(3)-XI**, CTEG01000046, [unknown aminoglycoside]					
YSMAA1_2_B7	**erm(X)**, X51472, [lincomycin, clindamycin, erythromycin, quinupristin , pristinamycin ia, virginiamycin s]					
YSMAA3_1_A4	**erm(X)**, X51472, [lincomycin, clindamycin, erythromycin, quinupristin , pristinamycin ia, virginiamycin s]	**aac(3)-XI**, CTEG01000046, [unknown aminoglycoside]				
YSMAA3_1_C3	**erm(X)**, U21300, [lincomycin, clindamycin, erythromycin, quinupristin , pristinamycin ia, virginiamycin s]					
YSMAA3_1_F3	**erm(X)**, U21300, [lincomycin, clindamycin, erythromycin, quinupristin , pristinamycin ia, virginiamycin s]	**erm(X)**, X51472, [lincomycin, clindamycin, erythromycin, quinupristin , pristinamycin ia, virginiamycin s]				
YSMAA3_1_G3	**erm(X)**, U21300, [lincomycin, clindamycin, erythromycin, quinupristin , pristinamycin ia, virginiamycin s]					
YSMAA4_1_B7	**erm(X)**, U21300, [lincomycin, clindamycin, erythromycin, quinupristin , pristinamycin ia, virginiamycin s]					
YSMAA4_1_E7	**erm(X)**, U21300, [lincomycin, clindamycin, erythromycin, quinupristin , pristinamycin ia, virginiamycin s]					
YSMAA5_1_B12	**erm(X)**, U21300, [lincomycin, clindamycin, erythromycin, quinupristin , pristinamycin ia, virginiamycin s]	**tet(Z)**, AF121000, [tetracycline, doxycycline]				
YSMAA5_1_F9	**erm(X)**, X51472, [lincomycin, clindamycin, erythromycin, quinupristin , pristinamycin ia, virginiamycin s]	**aac(3)-XI**, CTEG01000046, [unknown aminoglycoside]	**cmx**, U85507, [chloramphenicol]	**aph(6)-Id**, M28829, [streptomycin]	**aph(3")-Ib**, AF321551, [streptomycin]	**aph(3')-Ia**, X62115, [kanamycin, neomycin, lividomycin , paromomycin, ribostamycin]
YSMAA5_1_F11	**erm(X)**, U21300, [lincomycin, clindamycin, erythromycin, quinupristin , pristinamycin ia, virginiamycin s]	**ant(2")-Ia**, X04555, [gentamycin, tobramycin]				
YSMAA5_1_G11	**erm(X)**, U21300, [lincomycin, clindamycin, erythromycin, quinupristin , pristinamycin ia, virginiamycin s]	**ant(2")-Ia**, X04555, [gentamycin, tobramycin]				

Supplementary Table 6. Acquired resistance gene cassettes predicted to be present in the representative isolates using ResFinder.

Isolate	Clindamycin (10 µg)	Doxycycline (30 µg)	Penicillin G (1 µg)	Chloramphenicol (30 µg)	Vancomycin (30 µg)	Fosfomycin (200 µg)
YSMAA1_1_B1	-	-	-	-	-	+
YSMAA1_1_C1	-	-	-	-	-	+
YSMAA1_1_C6	-	-	-	-	-	+
YSMAA1_1_D6	-	-	-	-	-	+
YSMAA1_1_D8	-	-	-	-	-	+
YSMAA1_1_F5	-	-	-	-	-	+
YSMAA1_1_F6	+	-	-	-	-	+
YSMAA1_1_F7	-	-	+	-	-	+
YSMAA1_1_H1	-	-	-	-	-	+
YSMAA1_1_H4	-	-	-	-	-	+
YSMAA1_2_B5	-	-	-	-	-	+
YSMAA1_2_B7	+	-	-	-	-	+
YSMAA1_2_C5	-	-	-	-	-	+
YSMAA1_2_D10	-	-	-	-	-	+
YSMAA1_2_G9	-	-	-	-	-	+
YSMAA1_2_H3	-	-	-	-	-	+
YSMAA3_1_A4	+	-	-	-	-	+
YSMAA3_1_C3	+	-	-	-	-	+
YSMAA3_1_F3	+	-	-	-	-	+
YSMAA3_1_G3	-	-	-	-	-	+
YSMAA3_1_H1	-	-	-	-	-	+
YSMAA4_1_B7	+	-	-	-	-	+
YSMAA4_1_E7	+	-	-	-	-	+
YSMAA4_1_E8	-	-	-	-	-	+
YSMAA5_1_B12	+	-	-	-	-	+
YSMAA5_1_D11	-	-	-	-	-	+
YSMAA5_1_F9	-	-	-	+	-	+
YSMAA5_1_F11	+	-	-	-	-	+
YSMAA5_1_G11	+	-	-	-	-	+
YSMAA5_1_H11	-	-	-	-	-	+

Supplementary Table 7. Antibiotic susceptibility disc diffusion assay performed on the representative isolates using the EUCAST protocol. "+" refers to no visible inhibition around the disk (resistance phenotype) while "-" refers to a visible zone of clearing around the disk (susceptible phenotype).
